# Intimate partner violence in Nepal: Latent patterns and association with depressive symptoms

**DOI:** 10.1016/j.ssmph.2019.100481

**Published:** 2019-11-20

**Authors:** Cari Jo Clark, Yuk Fai Cheong, Jhumka Gupta, Gemma Ferguson, Binita Shrestha, Prabin Nanicha Shrestha, Kathryn M. Yount

**Affiliations:** a Hubert Department of Global Health, Rollins School of Public Health, Emory University, 1518 Clifton Road NE, Atlanta, GA, 30322, USA; b Department of Psychology, Emory University, Atlanta, GA, USA; c Department of Global and Community Health, College of Health and Human Services, George Mason University, 4400 University Drive, Fairfax, VA, 22030, USA; d 1001 Connecticut Avenue, NW Suite 909, Washington D.C, 20036, USA; e Department of Sociology, Emory College of Arts and Sciences, Emory University, 1555 Dickey Dr. 225 Tarbutton Hall, Atlanta, GA, 30322, USA

**Keywords:** Intimate partner violence, IPV, Domestic violence, Nepal, Latent class analysis

## Abstract

Existing data suggest that there are distinct patterns (or classes) of intimate partner violence (IPV) experience that depart from dichotomous categorizations used to monitor progress toward Sustainable Development Goal 5.2. Less is known about the patterning of IPV in non-Western settings. This study estimates distinct classes of IPV experience in Nepal and examines potential community-level variability in these classes and in the association between IPV class and depressive symptoms. This study used data collected in 2016 from a random sample of Nepalese married women of reproductive age (N = 1440) living in 72 communities in three districts (Nawalparasi, Chitwan, and Kapilvastu). We used fixed effects and random effects latent class models of 2 through 6 classes. We fit a negative binomial regression model adjusted for relevant confounders to examine the relationship of the latent IPV classes with depressive symptoms. A four-class model was the best fitting. It included a “low exposure” class (77.36% of the sample) characterized by a low probability of experiencing any form of IPV, a “sexual violence” class (9.03% of the sample) characterized by a high probability of experiencing a form of sexual violence, a “moderate violence” class (6.60% of the sample) characterized by modest probabilities of experiencing less severe emotional and physical IPV, and a “systematic violence” class (7.01% of the sample) characterized by a high probability of being exposed to all forms of IPV. Adding random effects did not improve model fit, suggesting no community-level variations in classes. Relative to membership in the low exposure class, membership in all other classes was associated with a higher count of depressive symptoms. Those in the systematic class had a mean weighted symptom count 2.29 times that of the low exposure group. Classes of IPV exposure must be identified to ensure that surveillance and programming are attuned to women's experiences of violence.

## Highlights

• First quantitative examination of classes in women's experience of IPV in Nepal.
• First multilevel LCA to identify common classes of IPV across communities.
• Classes of experience corroborate those found in other settings.
• Classes of experience are different from the way that IPV is monitored globally.

## 1 Introduction

Intimate partner violence (IPV) is a global health and development problem that affects nearly 1 in 3 women globally and throughout Asia (Devries et al., 2013). In Nepal, more than 1 in 4 women of reproductive age experience physical or sexual IPV in their lifetimes (Ministry of Health and Population (MOHP), 2012). Other research has found significant but varying estimates of the prevalence of women's experience of IPV in Nepal (Office of the Prime Minister and Council of Ministers Government of Nepal, 2012; Puri, Shah, & Tamang, 2010).

While prevalence estimates are useful to measure the magnitude of the problem and to monitor progress toward Sustainable Development Goal (SDG) 5.2, the elimination of violence against women and girls in public and private spheres (United Nations, 2015), they remain a crude measure of a multifaceted phenomenon. A consistent finding in IPV research globally is the co-occurrence of different forms of IPV (Garcia-Moreno, Jansen, Ellsberg, Heise, & Watts, 2006; Heise, 2012), suggesting a pattern of violent experiences that are unnaturally delineated as exposure to emotional, physical, or sexual IPV. Existing data, mostly from Western settings, suggest that there are distinct classes of IPV that do not conform to the dichotomous targets currently used to monitor progress toward SDG 5.2. Less is known about the alignment in non-Western settings. To date, no quantitative examination has been conducted on the possible patterning of South Asian women's experiences of IPV.

Understanding the nature of the violence women experience provides insights into the types of services that may be needed and potential differences across women with different patterns of exposure. In an environment where scarce resources must be allocated to both prevention and response, a more accurate understanding of the distribution of women across potentially distinct classes of IPV could facilitate a more efficient allocation of resources. Moreover, little is known about how distinct classes of IPV exposures may be associated (similarly or differentially) with poor mental health. Prior work examining latent classes of IPV has indicated that various measures of mental health vary with such patterns (Ansara & Hindin, 2011; Cale, Tzoumakis, Leclec, & Breckenridge, 2017; Carbone-López, Kruttschnitt, & Macmillan, 2006; Choi, Weston, & Temple, 2017; Dutton, Kaltman, Goodman, Weinfurt, & Vankos, 2005; Haynie et al., 2013; McNaughton Reyes, Maman, Chen, Groves, & Moodley, 2018; Weiss et al., 2017). Such research is lacking in Nepal, where data on mental health are scarce but suggestive of a notable mental health burden (Risal, Manandhar, Linde, Steiner, & Holen, 2016).

The present study begins to fill this gap using data from the baseline assessment of an IPV prevention trial in three districts in Nepal. Specifically, we: 1) examine the patterning of items measuring psychological, physical, and sexual IPV using latent class analysis; 2) test for community-level variation in this patterning; and 3) examine the relationship between membership in each IPV latent class and respondents’ reported number of depressive symptoms, a leading contributor to the global burden of disease (World Health Organization, 2017).

### 1.1 Latent class analysis and IPV

Latent class analysis is a latent, person-centered analytic approach whereby individuals can be classified into subgroups (or classes) based on their responses to measured variables, such as items measuring psychological, physical, and sexual IPV (Collins & Lanza, 2010). Prior latent class analysis and related latent analytic approaches have identified a range of IPV classes. In community samples, commonly identified classes include an unexposed category and a systematic abuse category, which is characterized by exposure to frequent and multiple types of IPV (Ansara & Hindin, 2010; Carbone-López et al., 2006; Choi et al., 2017; Grest, Lee, Gilreath, & Unger, 2018; Gupta et al., 2018; Haynie et al., 2013; Heise, 2012; Krishnakumar, Conroy, & Narine, 2018; Macmillan & Gartner, 1999; McNaughton Reyes, Maman, Chen, Groves, & Moodley, 2018; Spencer, Renner, & Clark, 2016; Watson & Parsons, 2005; Weiss et al., 2017). Other identified patterns are more diverse, with the degree of diversity depending in part on the number of types of IPV included and the number of unique items for each type. These other classes include one characterized mainly by psychological violence and controlling behaviors, one characterized by multiple but less severe forms of violence, and one characterized by sexual violence, among other less consistently identified classes (Ansara & Hindin, 2010; Beck, Anderson, O'Hara, & Benjamin, 2013; Cale et al., 2017; Carbone-López et al., 2006; Choi et al., 2017; Grest et al., 2018; Gupta et al., 2018; Haynie et al., 2013; Heise, 2012; Krishnakumar et al., 2018; Macmillan & Gartner, 1999; McNaughton Reyes, Maman, Chen, Groves, & Moodley, 2018; Watson & Parsons, 2005; Weiss et al., 2017).

When examined in relation to depressive symptoms or distress, the systematic violence class (or related designations of very severe violence) has consistently been associated with the greatest symptomatology (Ansara & Hindin, 2011; Cale et al., 2017; Carbone-López et al., 2006; Choi et al., 2017; Dutton et al., 2005; Haynie et al., 2013; McNaughton Reyes, Maman, Chen, Groves, & Moodley, 2018; Weiss et al., 2017). Other classes are more variably related to depressive symptoms, with the sexual violence class showing the most diverse pattern ranging from no association with symptomatology (Choi et al., 2017) to associations similar to other classes of violence experience, except systematic violence (Weiss et al., 2017). Therefore, context matters not only in the content of each class but also in relationship to poor mental health.

In the small but growing body of latent class analyses of IPV, a focus on women's experiences of IPV in non-Western settings is lacking. Almost all latent class analyses have been conducted among high-income Western populations, with unclear applicability to women in other socioeconomic and cultural settings. Few studies have examined these potential geographic differences (e.g. Heise, 2012) and none have examined them using multilevel analytic methods. A multilevel analysis can provide insight into contextual factors that might influence different patterns of IPV exposure. To our knowledge, no prior studies have used latent class analysis to explore patterns of IPV experience and potential mental health correlates among women in Asia. Therefore, this study begins to address these gaps using baseline data from an ongoing IPV prevention trial in three districts in Nepal.

### 1.2 IPV in Nepal

In Nepal, an estimated 26% of ever-married women have experienced psychological, physical, or sexual IPV (Ministry of Health, New ERA, & ICF, 2017). The estimate five years prior was 32%, suggesting a decreasing but still high prevalence over time. These apparent changes are occurring in a social and political landscape where lawmakers have taken measures to improve the status and inclusion of Nepali women, including making IPV a punishable offense, and gains include improved maternal and child health, women's inclusion in the political realm, and increased education for girls (Ghimire & Samuels, 2014; United Nations Development Programme, 2016). The Change Starts at Home Project (Change), a trial of a social and behavior change communication strategy to prevent IPV, is set within these broad economic, social, and political changes. In this analysis, we use baseline data from this trial to answer three research questions. First, are there distinct patterns that characterize women's experiences of IPV? Second, are there community-level differences in this patterning? Third, are the subgroups of IPV experience differentially associated with depressive symptoms?

## 2 Methods

### 2.1 Population and sample

The Change study has been underway since March 2016 in three districts of Nepal: Nawalparasi, Kapilvastu, and Chitwan. The populations in all three of these districts are more than 80% Hindu, but mean ages at first marriage, levels of female land ownership, and levels of female literacy vary (Central Bureau of Statistics [Nepal], 2013, 2014). These districts were selected because they are located in the *terai* region—where the estimated prevalence of IPV is highest (Ministry of Health et al., 2017). Within each district, 12 village development committees (VDCs), geographic subdivision of districts with governance responsibilities, were purposely selected. Eligible VDCs had to be at least 30–40 km in distance from one another and have separate major markets and major health centers. Within the VDC, two wards were selected randomly using probability proportionate to size sampling among eligible wards, defined as having a total household population between 100 and 550 (a size assumed appropriate for project activities). Simple random sampling using a sampling frame developed for the study was used to recruit 20 eligible female survey participants from each ward (age 18–49 years, husband at least age 18 years, reside regularly in the study area, and the wife and husband live together a majority of the year) for a total of 1440 participants. A total of 1982 individuals from 72 wards were assessed for eligibility, resulting in a response rate of 72.65% and a cooperation rate of 84.16% (Clark et al., 2019). Participants provided written informed consent, and the study was approved by the institutional review boards of the investigators’ institutions, the Nepal Health Research Council, and the District Development Committees representing the study sites.

### 2.2 Measures

Psychological, physical, and sexual IPV in the prior 12 months was assessed with standard items from the What Works to Prevent Violence Against Women and Girls consortium (Clark et al., 2019). These items were based closely on those administered in the World Health Organization Multi-country study on Women's Health and Violence Against Women (World Health Organization, 2005), which has been used in research all over the world and is very similar to the IPV scale used in the Demographic and Health Survey Domestic Violence Module, which has been administered to nationally representative samples of women of reproductive age in Nepal (Ministry of Health et al., 2017). The scale includes four psychological, five physical, and three sexual IPV items using behaviorally specific language. One psychological item (experiencing insults) was not included in the analysis because a prior latent class analysis on a large, multi-country dataset found that this item did not distinguish patterns of violence (Heise, Clark, Pallitto, & Garcia-Moreno, forthcoming). Cronbach's alpha for the measure was 0.92 in this sample. Table 1 provides the wording and the proportion of women reporting the occurrence within the prior 12 months for each item included in the latent class analysis.

**Table 1:** Items used to assess intimate partner violence, Nepal (N = 1440).

Psychological Intimate Partner Violence	Proportion (SD)
Belittled or humiliated in front of others	0.13 (0.34)
Scared or intimated	0.15 (0.36)
Threatened to hurt her/someone she cared about	0.07 (0.25)
**Physical Intimate Partner Violence**	
Pushed or shoved	0.15 (0.35)
Slapped or had an object thrown at her that could hurt	0.13 (0.34)
Hit you with a fist or something that could hurt	0.08 (0.27)
Kicked, dragged, beaten, choked or burnt	0.06 (0.24)
Threatened or actually used a weapon	0.03 (0.18)
**Sexual Intimate Partner Violence**	
Forced to have sex when did not want to	0.15 (0.36)
Had sex because afraid partner might become violent	0.16 (0.37)
Other unwanted sexual experiences	0.09 (0.29)

Note: Some variables contain up to 2 missing values.

Depressive symptoms were measured with the Patient Health Questionnaire-8, which had been validated previously in one of the study districts (Kohrt, Luitel, Acharya, & Jordans, 2016). Accordingly, the frequency (not at all [0], sometimes [1], usually [2], and always [3]) of occurrence of each depressive symptom was assessed in the prior 2 weeks, using a water glass graphic to assist in frequency assessment. A scale score was computed to represent a weighted symptom count ranging from 0 to 24 (Cronbach's alpha = 0.89 in this sample).

Several potential confounders of the relationship between IPV and depressive symptoms were also measured. Age was measured in years (range, 18–49 years). Educational attainment was measured categorically as none, primary, some secondary, and secondary School Leaving Certificate. Financial stress was measured with one item indicating whether the respondent reported that she or her husband frequently felt stressed because of not having enough income. Experience of maltreatment in childhood was measured with an item assessing the frequency (never, a few times, or often) with which the respondent was beaten as a child by a family member. Because of the small percentage of respondents (<2%) who reported being beaten often as a child, the measure was subsequently dichotomized as beaten as a child or not beaten as a child. Self-efficacy was measured with four items from the Generalized Self-Efficacy scale (Schwarzer & Jerusalem, 1995) that focused on solving problems with perseverance and, when opposed, dealing with unexpected events and handling what comes her way. The items were measured on a four-point scale assessing how accurately each response category reflected the respondent's belief in their capacities to cope with problems successfully (not at all true [1], hardly true [2], moderately true [3], and exactly true [4]), so that a sum of the scores ranged from 4 to 16 (Cronbach's alpha = 0.91 in this sample). Two additional scales were developed for the study to measure the quality of relationships with the natal and marital family, which are sources of stress or support. For each family type, the respondent was asked if she felt loved and cared for, could express her opinion freely on matters that are important to her, and if a member of her or her husband's family would tell her husband to stop if he physically hurt her. Answer choices were on a 4-point Likert scale from strongly disagree (0) to strongly agree (3), so that a sum of the scores ranged from 3 to 12 (Cronbach's alpha was 0.84 for natal family relations and 0.92 for in-law family relations in this sample).

### 2.3 Data analysis

Latent class analysis is a person-centered analytic strategy that allows us to identify patterns/classes of violence exposure based on respondents' responses to the items assessing IPV (Collins & Lanza, 2010). Classes that are identified are distinct from one another, but individuals within a class have similar responses to the items being analyzed. To account for the nested structure of the data and to examine the geographic variability in IPV patterning, we used multilevel and fixed effects latent class analysis (Asparouhov & Muthén, 2008; Henry & Muthén, 2010; Vermunt, 2008). We adopted a multistage approach, as postulated by Henry and Muthén (2010). First, we ran a series of fixed effects latent class models, including 2 through 6 classes, ignoring the clustered nature of the data. Multiple starts were used to protect against the identification of a local maxima for each model fit. We determined the best-fitting model based on the Bayesian Information Criterion (BIC) (Nylund, Asparouhov, & Muthén, 2007), the Vuong-Lo-Mendell-Rubin likelihood ratio test (to compare the fit of the model against one with one less category), and the principles of parsimony and interpretability (Collins & Lanza, 2010). Mean posterior probabilities (mean probability of classification into each class) and entropy (summary measure of the degree of certainty in the posterior classification) were then examined to ascertain the model's ability to categorize the study population into classes. We performed a sensitivity analysis to determine if the choice of best-fitting latent class model and the nature of the latent class (inspection of the posterior probabilities) differed by whether the insult item was included or not. The exclusion of this item did not alter either of these parameters and was not included in the analysis.

After determining the best-fitting fixed effects model, we used a one-factor parametric model (assuming normally distributed random means) and a nonparametric model with latent classes at the community level (Henry & Muthén, 2010) to assess the clustering effects. For the one-factor parametric model, the random factor represents combined latent within-ward latent class proportions (Finch & French, 2014). Such an approach reduces computational demands via dimensionality reduction of the random means for the latent means for the within-ward latent classes. We attempted a random effects model first, but it did not converge after a day and was discontinued. The nonparametric approach allows nonnormality in the distribution of the random means (Henry & Muthén, 2010). Finally, to examine the relationship of the latent IPV classes on depressive symptoms, we estimated a multilevel negative binomial regression model. A negative binomial model is an appropriate choice when the outcome measure is a count and it is overdispersed, meaning that it has greater variability than would be expected given the model (Cox, 1983). In the case of count data, under the Poisson model, the variance is assumed equal to the mean. As this assumption was not met (Table 2), the negative binomial model was used. To evaluate whether this estimation strategy was appropriate, overdispersion was assessed using the scaled Pearson statistic (value > 1 denotes overdispersion). The results of an unconditional model indicated limited variability in the log count of depressive symptoms across wards (_Ʈ_ = 0.05; z [71_df_] = 1.30; p = 0.10) and confirmed overdispersion of the depressive symptom count (scaled Pearson value = 3.98). Therefore, a single-level negative binomial regression model was fit to account for the distribution of the depressive symptoms. For ease of interpretation, we estimated the mean symptom counts for each class, holding all other variables at their mean (continuous variables) or null (dichotomous variables) values. For the latent variable models, we used Mplus version 8. For the negative binomial regression models, we used SAS version 9.4.

**Table 2:** Sample characteristics, Nepal (N = 1440).

	Individual
	Percent (No.)
Household financial stress^a^	45.16% (649)
Individual maltreated as a child	31.74% (457)
**Mean (SD)**	
Age	34.22 (8.31)
Respondent's educational level	1.33 (1.10)
Self-efficacy^a^	12.58 (3.37)
Natal-family relationship quality^a^	10.98 (1.82)
In-law relationship quality^a^	9.88 (2.48)
Depressive symptoms^a^	1.99 (3.05)

Note.

a Variable has up to 3 missing values.

## 3 Results

### 3.1 Characteristics of the study population

On average, the respondents were aged 34.22 years and had completed primary education (Table 2). Nearly half of the respondents reported household financial stress, and nearly one-third reported maltreatment as a child. Reported self-efficacy was high, averaging nearly 12.58 out of 16.00, suggesting strong problem solving and coping among women. Both natal- and marital-family relationships were reportedly strong, with mean scores of 10.98 and 9.88 out of 12.00, respectively. Finally, the weighted count of depressive symptoms was quite low, at 1.99 out of a total of 24.00.

### 3.2 Classes of exposure to IPV in the study population

Across all fixed effects models except the six-class solution (Table 3a), the Vuong-Lo-Mendell-Rubin likelihood ratio test was significant, suggesting that each larger model through the five-class solution was a better fit. Similarly, there is a clear improvement (decrease) in the BIC across all subsequent models, except for the six-class solution. However, the amount of improvement in the BIC diminishes steadily across models, suggesting more incremental gain after a three-class solution, with a change of only 10 between the four- and five-class solutions. As the four-class solution had strong posterior probabilities (0.89, 0.98, 0.95, and 0.98 for classes 1, 2, 3, and 4, respectively), entropy (0.93), and interpretability and was more parsimonious than the five-class model, it was chosen for further analysis. Adding random effects (parametrically and nonparametrically) made little difference in model fit or resulted in worse fit (Table 3b), suggesting little or no ward-level variation. Therefore, the fixed effects four-class solution was considered the final best-fitting model.

**Table 3a:** Fixed effects model fit two to six classes (N = 1440).

	Number of Classes of Exposure to Intimate Partner Violence				
	2	3	4	5	6
Free parameters	23	35	47	59	71
Log likelihood	−3725.80	−3422.98	−3311.21	−3262.54	−3227.00
Bayesian information criteria	7618.86	7100.50	6964.22	6954.16	6970.33
Entropy	0.95	0.91	0.93	0.93	0.92
Bootstrap likelihood ratio test *P*-value	<0.01	<0.01	<0.01	0.02	0.07

**Table 3b:** Random effects models for 4 latent classes (N = 1440).

Parametric Approach With Level 2 Factor on Random Latent Class Intercepts	
Free parameters	50
Log likelihood	−3299.85
Bayesian information criteria	6963.32
Entropy	0.92
**Nonparametric Approach-2 Between Classes**	
Free parameters	51
Log likelihood	−3299.24
Bayesian information criteria	6969.37
Entropy	0.80
**Nonparametric Approach-3 Between Classes**	
Free parameters	55
Log likelihood	−3296.31
Bayesian information criteria	6992.60
Entropy	0.76

**Fig. 1:**
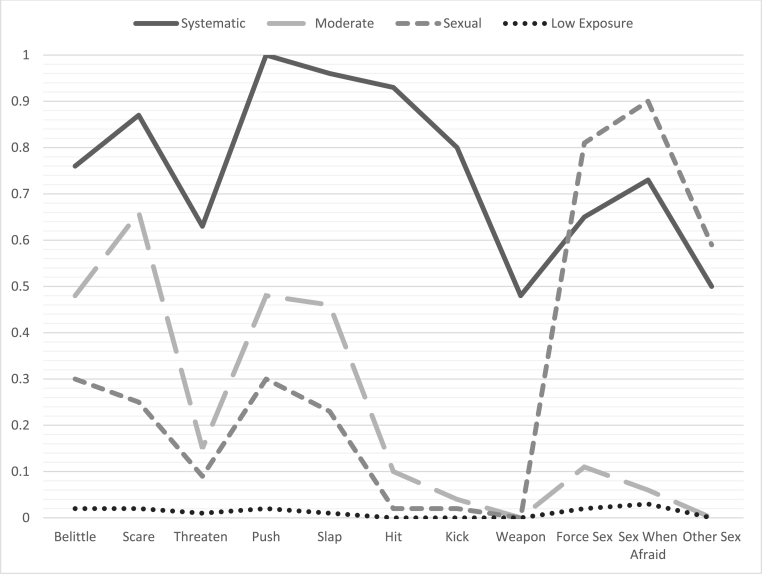
Probability of endorsing an item conditional on class.

Fig. 1 presents a profile plot that depicts the probability of endorsing an item, conditional on class. Class names were selected to be consistent with prior latent class analysis of IPV data to the extent possible. In the four-class fixed effects model, the most frequent class was the low exposure class, to which 77.36% of the sample belonged. This class was characterized by low probability of experiencing any of the behaviors assessed. The next most frequent class (9.03% of the sample) was the sexual violence class, which was characterized by a high probability of reporting forms of sexual violence (greater than 0.80 on forced sex and having sex out of fear). The other two classes were similar in size but captured different patterns of violence. The moderate violence class (6.60% of the sample) was characterized by modest probabilities of experiencing some form of emotional violence and less severe forms of physical violence, such as pushing and slapping and a low probability of experiencing severe forms of physical violence, defined as those likely to cause physical injury (Straus & Gelles, 1986) or any form of sexual violence. The remaining class systematic violence class (7.01% of the sample) was characterized by a high probability of being exposed to every form of violence assessed.

### 3.3 Classes of exposure to IPV and depressive symptoms

All classes were associated with a higher weighted symptom count relative to the low exposure class (Table 4), controlling for age, educational level, household financial stress, prior exposure to child maltreatment, self-efficacy, and natal and in-law family relationship quality. In post hoc analysis, all classes were significantly different from one another, except for the moderate and sexual violence classes. Individuals classified in the moderate violence class had a mean weighted depressive symptom count (mean = 5.34) that was 1.52 (exponential of 0.42) times that of the low exposure class. Those classified as experiencing sexual IPV had a mean weighted depressive symptom count (mean = 5.15) that was 1.45 (exponential of 0.37) times higher than those of the low exposure class. Those classified in the systematic violence class had a mean weighted symptom count (mean = 8.12) that was 2.29 (exponential of 0.83) times that of the low exposure group. Using a cut point of ≥10, which has been shown in research in Nepal to result in good sensitivity (0.94) and adequate specificity (0.80) (Kohrt et al., 2016), 3.1% (N = 45) of the entire sample would be considered depressive, which is similar to a prevalence estimate (3.5%) from a nationally representative nationwide survey of women aged 26–35 years in Nepal (Risal et al., 2016). Using this cut point, 21% (N = 21) of the respondents in the systematic violence class would be classified as depressed versus 5% (N = 6) of the respondents in the sexual violence class, 4% (N = 4) in the moderate violence class, and 1% (N = 14) in the low exposure class.

**Table 4:** Multivariate negative binomial regression model of the relationship between intimate partner violence (IPV), other covariates, and mean weighted count of depressive symptoms (N = 1433).

Parameter	Estimate	95% Confidence Interval		*P*-value
Intercept	1.50	0.85	2.15	<.0001
Age	0.01	−0.00	0.02	0.053
Education	−0.07	−0.15	0.01	0.103
Household financial stress	0.72	0.56	0.88	<.0001
Childhood maltreatment	0.20	0.03	0.37	0.023
Self-efficacy	−0.06	−0.08	−0.04	<.0001
Natal family relationship	−0.03	−0.07	0.01	0.183
In-law family relationship	−0.08	−0.12	−0.05	<.0001
Low exposure	ref			
Moderate violence	0.42	0.11	0.73	0.008
Sexual violence	0.37	0.10	0.65	0.007
Systematic violence	0.83	0.53	1.13	<.0001

## 4 Discussion

This study was the first of its kind in Nepal and, more broadly, in South Asia to investigate potential classes of women in terms of their exposure to IPV. This study answered three important questions. First, are there distinct patterns that resemble women's experience of IPV? Second, are there community-level differences in this patterning? Third, are the subgroups of IPV experience differentially associated with depressive symptoms? Answers to these questions offer insights to strengthen and target health and development programming and have implications for existing IPV surveillance measures.

### 4.1 IPV classes

Findings from this study revealed four subgroups of IPV exposure reported by women in Nepal. Although no purely psychological violence category was identified, the identified classes were similar to prior research including an unexposed/low exposure class, a systematic violence class, a class characterized by multiple but less severe forms of violence; and a class characterized by sexual violence (Ansara & Hindin, 2010; Beck et al., 2013; Cale et al., 2017; Carbone-López et al., 2006; Choi et al., 2017; Grest et al., 2018; Gupta et al., 2018; Heise, 2012; Krishnakumar et al., 2018; Macmillan & Gartner, 1999; McNaughton Reyes, Maman, Chen, Groves, & Moodley, 2018; Spencer et al., 2016; Watson & Parsons, 2005; Weiss et al., 2017). These findings suggest that there is classifiable diversity in women's experiences of IPV that may be somewhat similar across geographic, national, and sociocultural settings. Further, the lack of a psychological violence class suggests a high co-occurrence of different types of IPV. Therefore, modeling the different forms of IPV separately as psychological, physical, and sexual obscures the underlying patterns that cut across types.

Second, community-level differences in this patterning were not apparent. Despite considerable diversity in Nepal (Zwi, Garfield, & Loretti, 2002), the identified four-class solution did not vary by community, suggesting a consistent patterning of IPV at least within the 72 wards across three districts in Nepal. Although this finding is somewhat different from the prior multi-country latent class analysis study of sites in Africa and Latin America (Heise, 2012), differences found in the multi-country study pertained mostly to the presence or absence of a category not entirely different patterns, suggesting that broad patterns are discernible even across or within geographic, national, and sociocultural settings.

### 4.2 Implications for mental health

Differences were detectable across IPV subgroups in relation to depressive symptoms. The association of depressive symptoms with IPV experience was especially strong in the systematic IPV subgroup, within which approximately 1 in 5 women might be considered to have depression, which is 4 times the prevalence of depression found in prior research among a random, nationwide sample of women aged 18–65 years in Nepal (5.4%) and 5 times the estimate for women aged 26–35 years (3.5%) (Risal et al., 2016). The elevated mental health impact of IPV found in this study is similar to the majority of prior latent and cluster analyses of IPV and depressive symptoms (Ansara & Hindin, 2011; Cale et al., 2017; Carbone-López et al., 2006; Choi et al., 2017; Dutton et al., 2005; Haynie et al., 2013; McNaughton Reyes, Maman, Chen, Groves, & Moodley, 2018; Weiss et al., 2017). The presence of this distinct, very severe class corroborates research using latent methods to investigate IPV along with other forms of violence. The most severe categories in much of this related research consisted of high probabilities of different forms of violence in addition to IPV (Garthe, Sullivan, & Behrhorst, 2018; Golder, Connell, & Sullivan, 2012; Sipsma et al., 2015), highlighting the significant mental health consequences of polyvictimization, whether defined as exposure to multiple types of one form of violence or to multiple forms of violence (Contractor, Caldas, Fletcher, Shea, & Armour, 2018). The similarity in the magnitude of the relationship between depressive symptoms and both the sexual violence and moderate violence classes is in alignment with prior, albeit limited, research on adult IPV survivors (Weiss et al., 2017) but also contrary to the equally scarce research that identified no significant relationship between the sexual violence class and depressive symptoms (Choi et al., 2017). This latter study was conducted among US adolescents whose exposure to sexual violence was quite different from that of married women in the present study and the adult IPV survivors in prior research (Weiss et al., 2017). Overall, the presence of elevated symptoms of depression among women in all violence exposure subgroups echoes extensive prior research on the mental health correlates of IPV (Stewart, Vigod, & Riazantseva, 2016).

### 4.3 Implications for surveillance

Progress toward the elimination of all forms of violence against women and girls (SDG 5.2) will be accomplished, in part, with dichotomous measures of the proportion of girls and women aged 15 years or older who have experienced physical, sexual, or psychological IPV in the prior 12 months by form of violence and age (United Nations, 2015). Evidence from the present study, and others before, suggests that there are distinct patterns of IPV that commonly co-occur. In our study, none of the identified classes corresponds closely, except perhaps for the sexual abuse category, to the dichotomous SDG targets by IPV type, and some of the classes suggest different degrees of severity. Progress may be made but completely undetectable with the current formulation of the SDG targets, which require all measured acts of a particular form of violence to be eliminated before progress in that particular target indicator is evident; however, progress may be made within and across patterns that comprise acts from different types of violence before the elimination of all acts of a particular type of violence. Although the goal of the SDG is to eliminate all forms of violence against women and girls, the visibility of progress is an essential part of the surveillance mission to help align policy and associated investments. At present, the exact nature of the patterns depends in part on the number of items, the range of forms of IPV assessed, and the naming of the classes, which varies considerably in the literature. Future latent class analysis research relying on the most common measures of IPV may provide further insight on the patterns of IPV that are consistent across settings, to inform how to best capture the patterning of these classes of violence exposures for surveillance purposes. Finally, the graded association of IPV with depressive symptoms across classes suggest that designations of severity might best be made on the pattern and not on the occurrence of a particular act or type of IPV.

### 4.4 Implications for IPV prevention and response

The extreme scope of IPV in the systematic class suggests that responses for women in this subgroup are different in content and possibly mode of delivery than those experienced by women in the moderate and sexual violence classes. Because violence severity is a key predictor of formal service use (Goodson & Hayes, 2018), women in the systematic violence category are more likely than women in the other violence classes to seek psychosocial, medical, and legal resources. These response options are lifesaving when they are available and effective. However, they are often also the least utilized (Goodson & Hayes, 2018), including in Nepal (Ministry of Health et al., 2017), as they are frequently stigmatized and entail considerable social and financial repercussions, even when they are effective. Ongoing efforts by the Government of Nepal and numerous implementing organizations with funding from the United Kingdom Department for International Development are working towards this goal by improving security and access to justice (United Kingdom Department for International Development, 2018). The fact that the systematic violence category is among the most consistently identified across sociocultural settings suggests that the support of effective, safe, culturally appropriate options for women experiencing severe violence remains an area in need of continued support.

The programming needs of the other two classes (moderate violence and sexual violence) are potentially somewhat similar in key respects, as they both exemplify a use of violence that is more normative (i.e., undergirded by patriarchal gender norms). Manifestations of these norms include significant percentages of men and women in countries throughout the world, including in Nepal (Ministry of Health et al., 2017), who justify a man’s use of violence against his wife for some perceived transgression. More severe or lethal forms of violence, however, are more often negatively socially sanctioned (Brown, 1992; Counts, Brown, & Campbell, 1999), suggesting that there are limits to what is acceptable or justifiable. The lack of severe forms of violence in the moderate violence class fits this pattern. Similarly, a man's entitlement to sexual intercourse from his wife features prominently within the institution of marriage historically and cross-culturally (Yllö, 2016), which likely influences the size of the sexual violence class. Similarly, the United Nations (UN) Multi-country Study on Men and Violence in Asia and the Pacific found sites where significant proportions of men reported perpetrating sexual violence only (Fulu, Jewkes, Roselli, Garcia-Moreno, Men, & Violence research, 2013), although Nepal was not included in that study so direct comparisons are not possible. The presence of the sexual violence class was explained in part by gender norms that support sexual entitlement and sexual control over women (Fulu et al., 2013). The normative underpinnings of these two classes suggest the need for broad normative change and an emphasis on primary and secondary prevention. However, the UN Multi-country Study also found that perpetrators of partner sexual violence only share many risk factors with perpetrators of nonpartner sexual violence and nonfamilial interpersonal violence, suggesting the potential need for more specialized interventions for such perpetrators (Fulu et al., 2013). Although the present study cannot answer this question directly, the presence of distinct patterns of victimization underscore the need for clear understanding of the patterns for closely tailored prevention and response programming. Further analyses examining potential differences in help-seeking and the accessibility of natal and in-law supports across different exposure patterns could contribute valuable preliminary information towards more targeted responses. Furthermore, future IPV prevention and intervention research should examine the presence of any treatment heterogeneity by latent class, such as the classes identified herein. This may be particularly important for interventions that seek to reduce IPV by challenging social norms.

### 4.5 Limitations

Interpretation of the findings requires consideration of the study's limitations. First, this is a cross-sectional analysis; therefore, causality cannot be determined. The study does not have data on nonparticipants to assess whether a lack of participation was associated with key study variables. The internal coherence of the results and the consistency of the findings with prior research suggests that nonparticipation has not hindered our ability to answer the stated research questions. The use of latent class analysis is more complex than creating an IPV score as a simple sum across items, making it a less accessible approach to modeling IPV. However, classes of women's experience, especially sexual violence, would be obscured by a simple sum highlighting the importance of understanding distinct patterns of exposure. Our measure of IPV is standard among those in the consortium and is similar to that used for IPV surveillance in countries throughout the world. Nevertheless, the measure contains a small number of items that had to be dichotomized for analysis, which limits understanding of item frequency. The small number of items per form of violence potentially limits our ability to detect classes, such as a psychological violence only class; however, the lack of a psychological violence category may also simply reflect the considerable overlap in these experiences with physical and sexual IPV. The sample size is limited relative to the number of items that were included in the analysis. A larger sample would confer greater confidence in the classes and allow the inclusion of item frequency. And although many communities were included in the analysis and patterns identified are similar to that found in other settings, the study was limited to communities in three districts in Nepal. The presence of classes similar to those in other settings and the lack of community-level variability in the classes suggests that the findings are not likely driven by characteristics of the study communities. However, extrapolation beyond the study communities is not warranted given the sampling design. The latent class analysis classes are statistical constructs, and additional research, including qualitative research, is needed to examine the extent to which the patterns identified herein are consistent with women's lived experiences of IPV.

### 4.6 Conclusion

Study findings underscore the need for typologies of violence that clarify women's lived experiences of IPV. Such typologies will improve our capacity for surveillance and for aligning programs to women's needs. In a country and region with a high concentration of development initiatives, most of which contain at least an element of gender-focused programming, data that more closely capture the distinct nature of women's experiences is essential to implementing more effective health and development programming.

## Ethics statement

IRB approval has been obtained from the University of Minnesota (where the PI was at the time of grant receipt), Emory University (where the PI is currently located), George Mason University (professional home of a co-investigator), and the Nepal Health Research Council.

## Funding

This manuscript has been funded by a grant (#P06254) from UK aid from the UK government, via the What Works to Prevent Violence Against Women and Girls? Global Programme (www.whatworks.co.za). The funds were managed by the South African Medical Research Council. The views expressed do not necessarily reflect the UK government's official policies.

## Declaration of competing interest

None.

## References

[bib1] Ansara D.L., Hindin M.J. (2010). Exploring gender differences in the patterns of intimate partner violence in Canada: A latent class approach. Journal of Epidemiology & Community Health.

[bib2] Ansara D.L., Hindin M.J. (2011). Psychosocial consequences of intimate partner violence for women and men in Canada. Journal of Interpersonal Violence.

[bib3] Asparouhov T., Muthén B., Hancock G., Samuelsen K. (2008). Multilevel mixture models. Advances in latent variable mixture models.

[bib4] Beck C.J., Anderson E.R., O'Hara K.L., Benjamin G.A. (2013). Patterns of intimate partner violence in a large, epidemiological sample of divorcing couples. Journal of Family Psychology.

[bib5] Brown J.K., Brown J.K., Campbell J.C. (1992). Introduction: Definitions, assumptions, themes, and issues. Dorothy ayers counts.

[bib6] Cale J., Tzoumakis S., Leclec B., Breckenridge J. (2017). Patterns of intimate partner violence victimization among austrial and New Zealand female university students: An initial examination of child maltreatment and self-reported depressive symtpoms across profiles. Australian and New Zealand Journal of Criminology.

[bib7] Carbone-López K., Kruttschnitt C., Macmillan R. (2006). Patterns of intimate partner violence and their associations with physical health, psychological distress, and substance use. Public Health Reports.

[bib8] Central Bureau of Statistics [Nepal] (2013). Statistical year book of Nepal-2013.

[bib9] Central Bureau of Statistics [Nepal] (2014). Population atlas of Nepal.

[bib10] Choi H.J., Weston R., Temple J.R. (2017). A three-step latent class Analysis to identify how different patterns of teen dating violence and psychosocial factors influence mental health. Journal of Youth and Adolescence.

[bib11] Clark C.J., McGhee S., Ferguson G., Shrestha B., Shrestha P.N., Oakes J.M. (2019). Change Starts at Home: Baseline report of a trial to prevent intimate partner violence among married couples in Nepal.

[bib12] Collins L.M., Lanza S.T. (2010). Latent class and latent transition analysis with applications in the social, behavioral, and health sciences.

[bib13] Contractor A.A., Caldas S., Fletcher S., Shea M.T., Armour C. (2018). Empirically derived lifespan polytraumatization typologies: A systematic review. Journal of Clinical Psychology.

[bib14] Counts D.A., Brown J.K., Campbell J.C. (1999). To have and to hit: Cultural perspectives on wife beating.

[bib15] Cox D.R. (1983). Some remarks on overdispersion. Biometrika.

[bib16] Devries K.M., Mak J.Y., Garcia-Moreno C., Petzold M., Child J.C., Falder G. (2013). Global health. The global prevalence of intimate partner violence against women. Science.

[bib17] Dutton M.A., Kaltman S., Goodman L.A., Weinfurt K., Vankos N. (2005). Patterns of intimate partner violence: Correlates and outcomes. Violence & Victims.

[bib18] Finch W.H., French B.F. (2014). Multilevel latent class analysis: Parametric and nonparametric models. The Journal of Experimental Education.

[bib19] Fulu E., Jewkes R., Roselli T., Garcia-Moreno C., UN Multi-country Cross-sectional Study on Men and Violence research team (2013). Prevalence of and factors associated with male perpetration of intimate partner violence: Findings from the UN multi-country cross-sectional study on men and violence in Asia and the pacific. Lancet Glob Health.

[bib20] Garcia-Moreno C., Jansen H.A., Ellsberg M., Heise L., Watts C.H. (2006). Prevalence of intimate partner violence: Findings from the WHO multi-country study on women's health and domestic violence. Lancet.

[bib21] Garthe R.C., Sullivan T.N., Behrhorst K.L. (2018). A latent class Analysis of early adolescent peer and dating violence: Associations with symptoms of depression and anxiety. Journal of Interpersonal Violence.

[bib22] Ghimire A., Samuels F. (2014). Change and continuity in social norms and practices around marriage and education in Nepal. Country Report.

[bib23] Golder S., Connell C.M., Sullivan T.P. (2012). Psychological distress and substance use among community-recruited women currently victimized by intimate partners: A latent class analysis and examination of between-class differences. Violence Against Women.

[bib24] Goodson A., Hayes B. (2018). Help-seeking behaviors of intimate partner violence victims: A cross-national analysis in developing nations. Journal of Interpersonal Violence.

[bib25] Grest C.V., Lee J.O., Gilreath T., Unger J.B. (2018). Latent class Analysis of intimate partner violence perpetration and victimization among latino emerging adults. Journal of Youth and Adolescence.

[bib26] Gupta J., Willie T.C., Harris C., Campos P.A., Falb K.L., Garcia Moreno C. (2018). Intimate partner violence against low-income women in Mexico city and associations with work-related disruptions: A latent class analysis using cross-sectional data. Journal of Epidemiology & Community Health.

[bib27] Haynie D.L., Farhat T., Brooks-Russell A., Wang J., Barbieri B., Iannotti R.J. (2013). Dating violence perpetration and victimization among U.S. Adolescents: Prevalence, patterns, and associations with health complaints and substance use. Journal of Adolescent Health.

[bib28] Heise L.L. (2012). Determinants of partner violence in low and middle-income countries: Exploring variation in individual and population level risk london School of hygiene and tropical medicine.

[bib29] Heise, L. L., Clark, C. J., Pallitto, C., & Garcia-Moreno, C. (forthcoming). Using existing data to create a cross cultural measure of psychological partner aggression for use in monitoring the Sustainable Development Goals (SDGs). SSM-Population Health. 10.1016/j.ssmph.2019.100377 PMC6978474 31993478

[bib30] Henry K.L., Muthén B.O. (2010). Multilevel latent class Analysis: An application of adolescent smoking typologies with individual and contextual predictors. Structural Equation Modeling.

[bib31] Kohrt B.A., Luitel N.P., Acharya P., Jordans M.J. (2016). Detection of depression in low resource settings: Validation of the patient health Questionnaire (PHQ-9) and cultural concepts of distress in Nepal. BMC Psychiatry.

[bib32] Krishnakumar A., Conroy N., Narine L. (2018). Correlates of sex-specific yount adult college student dating violence typologies: A latent class analysis approach. Psychol Violence.

[bib33] Macmillan R., Gartner R. (1999). When she brings home the bacon: Labor-force participation and the risk of spousal violence against women. Journal of Marriage and Family.

[bib35] Ministry of Health, New ERA, & ICF (2017). Nepal demographic and health survey 2016.

[bib34] McNaughton Reyes H., Maman S., Chen M., Groves A., Moodley D. (2018). Patterns of intimate partner violence victimization among South African women and their relation to emotional distress during pregnancy and postpartum. Journal of Interpersonal Violence.

[bib36] ICF International Inc, Ministry of Health and Population (MOHP) [Nepal], New Era (2012). Nepal demographic and health survey 2011.

[bib37] Nylund K.L., Asparouhov T., Muthén B.O. (2007). Deciding on the number of classes in latent class Analysis and growth mixture modeling: A Monte Carlo simulation study. Structural Equation Modeling.

[bib38] Office of the Prime Minister and Council of Ministers Government of Nepal (2012). A study on gender-based violence conducted in selected rural districts of Nepal.

[bib39] Puri M., Shah I., Tamang J. (2010). Exploring the nature and reasons for sexual violence within marriage among young women in Nepal. Journal of Interpersonal Violence.

[bib40] Risal A., Manandhar K., Linde M., Steiner T.J., Holen A. (2016). Anxiety and depression in Nepal: Prevalence, comorbidity and associations. BMC Psychiatry.

[bib41] Schwarzer R., Jerusalem M., Weinman S.W.J., Johnston M. (1995). Generalized self efficacy scale. Measures in health psychology: A user's portfolio.

[bib42] Sipsma H.L., Falb K.L., Willie T., Bradley E.H., Bienkowski L., Meerdink N. (2015). Violence against Congolese refugee women in Rwanda and mental health: A cross-sectional study using latent class analysis. BMJ Open.

[bib43] Spencer R.A., Renner L.M., Clark C.J. (2016). Patterns of dating violence perpetration and victimization in U.S. Young adult males and females. Journal of Interpersonal Violence.

[bib44] Stewart D.E., Vigod S., Riazantseva E. (2016). New developments in intimate partner violence and management of its mental health sequelae. Current Psychiatry Reports.

[bib45] Straus M.A., Gelles R.T. (1986). Societal change and change in family violence from 1975 to 1985 as revealed by two national surveys. Journal of Marriage and Family.

[bib46] United Kingdom Department for International Development (2018). Integrated Programme for strengthening security and justice.

[bib47] United Nations (2015). Transforming our world: The 2030 agenda for sustainable development.

[bib48] United Nations Development Programme (2016). Asia-Pacific human development report. Shaping the future.

[bib49] Vermunt J. (2008). Latent class and finite mixture models for multilevel data sets. Statistical Methods in Medical Research.

[bib50] Watson D., Parsons S. (2005). Domestic abuse of women and men in Ireland: Report on the national study of domestic abuse.

[bib51] Weiss N.H., Dixon-Gordon K.L., Peasant C., Jaquier V., Johnson C., Sullivan T.P. (2017). A latent profile Analysis of intimate partner victimization and aggression and examination of between-class differences in psychopathology symptoms and risky behaviors. Psychological Trauma: Theory, Research, Practice and Policy.

[bib52] World Health Organization (2005). WHO multi-country study on women's health and domestic violence against women: Summary report of initial results onprevalence, health outcomes and women's responses.

[bib53] World Health Organization (2017). Depression and other common mental disorders: Global health estimates.

[bib54] Yllö K., Yllö K., Torres M.G. (2016). Prologue: Understanding marital rape in global context. Marital rape: Consent, marriage, and social change in global context.

[bib55] Zwi A.B., Garfield R., Loretti A., Krug E.G., Dahlberg L.L., Mercy J.A., Zwi A.B., Lozano R. (2002). Collective violence. World report on violence and health.

